# Supplementary material for: Hybrid Models and Biological Model Reduction with PyDSTool
Source: PLoS Comput Biol. 2012 Aug 9;8(8):e1002628. doi: 10.1371/journal.pcbi.1002628 (PMC3415397; doi:10.1371/journal.pcbi.1002628)
Supplement: Text S4 — Complete source code for the PyDSTool package (version 0.88.120504). Includes API documentation and help files linking to web pages. This file is identical to the current public release on Sourceforge.net. (ZIP) [file pcbi.1002628.s004.zip › PyDSTool/html/PyDSTool.MProject.MReg-class.html]

xml version="1.0" encoding="ascii"?


PyDSTool.MProject.MReg


| Home | Trees | Indices | Help | PyDSTool |
| --- | --- | --- | --- | --- |

Package PyDSTool ::
Module MProject ::
Class MReg

[hide private]

[frames] | no frames]

# Class MReg

source code

```
object --+
         |
        MReg
```

---

Registry class for Model descriptors and instances in Model projects.
For internal use by PyDSTool.


|  |  |
| --- | --- |
| Instance Methods | [hide private] |

|  |  |
| --- | --- |
| \_\_init\_\_(self)  x.\_\_init\_\_(...) initializes x; see x.\_\_class\_\_.\_\_doc\_\_ for signature | source code |

|  |  |
| --- | --- |
| add(self, descriptor)  descriptor expected to be an MDescriptor object. | source code |

|  |  |
| --- | --- |
| \_\_setitem\_\_(self, name, model\_instance) | source code |

|  |  |
| --- | --- |
| \_\_contains\_\_(self, name) | source code |

|  |  |
| --- | --- |
| \_\_getitem\_\_(self, name) | source code |

|  |  |
| --- | --- |
| \_\_delitem\_\_(self, name) | source code |

|  |  |
| --- | --- |
| remove(self, name) | source code |

|  |  |
| --- | --- |
| query(self, querykey, value)  Return info about stored model specifications. | source code |

**Inherited from `object`**:
`__delattr__`,
`__getattribute__`,
`__hash__`,
`__new__`,
`__reduce__`,
`__reduce_ex__`,
`__repr__`,
`__setattr__`,
`__str__`


|  |  |
| --- | --- |
| Properties | [hide private] |

**Inherited from `object`**:
`__class__`


|  |  |
| --- | --- |
| Method Details | [hide private] |

|  |  |
| --- | --- |
| \_\_init\_\_(self)  *(Constructor)* | source code |

x.\_\_init\_\_(...) initializes x; see x.\_\_class\_\_.\_\_doc\_\_ for
signature

Overrides: object.\_\_init\_\_: *(inherited documentation)*

|  |  |
| --- | --- |
| query(self, querykey, value) | source code |

Return info about stored model specifications. Valid query keys:
'orig\_name', 'in\_description'

  


| Home | Trees | Indices | Help | PyDSTool |
| --- | --- | --- | --- | --- |

|  |  |
| --- | --- |
| Generated by Epydoc 3.0.1 on Fri May 4 15:24:06 2012 | http://epydoc.sourceforge.net |
